# Investigation of the Mechanism of Zishen Yutai Pills on Polycystic Ovary Syndrome: A Network Pharmacology and Molecular Docking Approach

**DOI:** 10.1155/2021/6843828

**Published:** 2021-12-16

**Authors:** Yingyin Chen, Xinyi Chai, Ying Zhao, Xinqian Yang, Caiting Zhong, Yihui Feng

**Affiliations:** ^1^Guangzhou University of Chinese Medicine, Guangzhou 510405, China; ^2^Lingnan Medical Research Center, Guangzhou University of Chinese Medicine, Guangzhou 510405, China; ^3^Department of Gynecology, First Affiliated Hospital of Guangzhou University of Chinese Medicine, Guangzhou 510405, China; ^4^First Clinical Medical College of Guangzhou University of Chinese Medicine, Guangzhou 510405, China

## Abstract

**Background:**

Zishen Yutai Pills (ZSYTP) is a prescription based on traditional Chinese medicine used to treat kidney-deficient pattern in traditional Chinese medicine. It is also widely used clinically for the treatment of polycystic ovary syndrome (PCOS) with positive results. This study aims to explore the potential pharmacological mechanism of ZSYTP for the treatment of PCOS by a network pharmacology approach.

**Methods:**

Compounds were collected from the Traditional Chinese Medicine Systems Pharmacology Database and Analysis Platform and Bioinformatics Analysis Tool for Molecular mechanism of Traditional Chinese Medicine and TCM Database@ Taiwan, and the corresponding targets were retrieved from PubChem, Swiss Target Prediction, STITCH, and DrugBank. Meanwhile, PCOS targets were retrieved from the GeneCards database, the Online Mendelian Inheritance in Man database, National Center for Biotechnology Information Database, and DrugBank. Subsequently, multiple network construction and gene enrichment analyses were conducted with Cytoscape 3.8.2 software. Based on the previous results in the study, molecular docking simulations were done.

**Results:**

205 active compounds and 478 ZSYTP target genes were obtained after screening by ADME consideration. 1881 disease-related targets were obtained after removing duplicates. 148 intersection target genes between drug and disease targets were isolated. Gene ontology enrichment analysis and Kyoto Encyclopedia of Genes and Genomes analysis highlighted multiple gene functions and different signaling pathways to treat PCOS. Further molecular docking demonstrated the practicality of in vivo action of ZSYTP to a certain extent.

**Conclusions:**

It is possible that the pharmacological effect of ZSYTP on PCOS is linked to the hypoxia-inducible factor 1 (HIF-1) signaling pathway, improving insulin resistance, the variation on gene expression such as RNA splicing, and regulation of mRNA metabolic process. This study paves the way for further research investigating its mechanisms.

## 1. Background

Polycystic ovary syndrome (PCOS) is a common gynecological disorder affecting 5%–15% of women of reproductive age from all over the world [1]. Multiple aspects of a women's holistic health and its long-term effects are affected by PCOS. As one of the most common endocrine-reproductive-metabolic disorders, it affects reproductivity in females and is linked to endocrine disorders like insulin resistance and type 2 diabetes. PCOS patients are known to have an increased risk of cardiovascular disorders such as atherogenic dyslipidemia syndrome, coronary heart disease, and cerebrovascular morbidity. Beyond that, it also influences PCOS patients' mental health [2].

At present, due to the relatively unknown etiology of PCOS, there is no effective treatment protocol. Symptomatic treatment is mainly used in clinical practice, and long-term health management is needed. Traditional Chinese medicine (TCM) has long been utilized to treat infertility for centuries and has been proven effective in managing the symptoms of PCOS patients and promoting ovulation. In accordance with TCM theory, PCOS patients who have obesity or insulin resistance [3] can be widely categorized into several TCM patterns, kidney deficiency and phlegm, kidney deficiency and blood stasis, and kidney deficiency or insufficiency of both the spleen and the kidney [4]. It can be observed that most PCOS patients are of a kidney-deficient pattern. Zishen Yutai Pill (ZSYTP) is a patented formula used clinically for kidney-deficient-pattern patients [5]. Further studies have also shown that ZSYTP improved ovulatory functions in patients of this pattern. While the clinical efficacies of ZSYTP have been proven with multiple studies [6], the pharmacological mechanism of its therapeutic effect remains unclear.

In recent years, increasing research has been done on traditional or patented TCM formulae, and it can be a gift for drug discovery [7]. For instance, the study of the known TCM herb *Artemisia carvifolia* led to the discovery of artemisinin and subsequently became crucial in the fight against malaria [8].

A network pharmacology approach was chosen to uncover ZSYTP's pharmacological mechanism on PCOS, aiming to provide a protocol for studying patented formulae and a specific disease. This study also would provide the data and direction for further clinical trials to develop, improving the efficacy of future drug development studies.

## 2. Methods and Materials

### 2.1. Data Preparation

#### 2.1.1. Screening for Chemical Compounds of Each Herb in ZSYTP

Chemical compounds from 15 herbs in ZSYTP were obtained from the Traditional Chinese Medicine System Pharmacology Database [9] (TCMSP™, https://old.tcmsp-e.com/tcmsp.php), a unique systemic pharmacology platform devised for Chinese herbal medicines.

The relationships between drugs, targets, and diseases were obtained from a Bioinformatics Analysis Tool for Molecular mechanism of Traditional Chinese Medicine [10] (BATMAN-TCM, http://bionet.ncpsb.org.cn/batman-tcm/), which is a bioinformatics analysis tool specially designed for the research of the molecular mechanism of TCM and TCM Database@ Taiwan [11] (http://tcm.cmu.edu.tw/zh-tw/), the world's largest noncommercial TCM database.

Screening filters used in this study were OB ≥ 30% and DL ≥ 0.18 to maximize drug discovery. These active compounds were included in accordance with ADME consideration (Adsorption, Distribution, Metabolism, Excretion) which could ensure higher efficiency to the active compounds selected.

#### 2.1.2. Screening Target Genes of Each Herb in ZSYTP

We collated IUPAC International Chemical Identifier (InChi) and Canonical SMILES of each 205 chemical compounds of ZSYTP from PubChem database [12] (http://pubchem.ncbi.nlm.nih.gov/) to corroborate the uniqueness of these molecules in the database we built.

To predict interactions between chemicals and proteins, we input all molecular information of the 205 active compounds into STITCH [13] (http://stitch.embl.de) and Swiss Target Prediction [14] (http://www.swisstargetprediction.ch/index.php). PubChem database was utilized to provide drug-target identification. Finally, we found information on known drugs and their corresponding target genes in DrugBank [15](https://go.drugbank.com/), which is a pharmaceutical knowledge base.

#### 2.1.3. PCOS Targets Database Building

“Polycystic Ovary Syndrome” is the keyword used to retrieve relevant action targets. To obtain more comprehensive disease-related targets, 3 online databases were utilized, including the GeneCards database [16] (http://www.genecards.org/), the Online Mendelian Inheritance in Man database [17] (OMIM, http://www.omim.org/), National Center for Biotechnology Information database [18] (NCBI, https://www.ncbi.nlm.nih.gov/), and DrugBank database (http://www.drugbank.ca/). GeneCards, OMIM, and NCBI database provide comprehensive data on predicted and annotated human genotypes, while DurgBank offers data on the disease and the drugs available to it. Due to the nonstandard naming of the proteins collated from the 3 databases, we used the UniProt database [19] (http://www.uniprot.org/) to match the standard gene names.

### 2.2. Network Construction

#### 2.2.1. Network Construction Method

The network construction was built as follows: (1) ZSYTP active compound-potential target network (C-T network), (2) PPI network of PCOS disease targets, (3) PPI network of ZSYTP drug targets, and (4) PPI network of interaction between PCOS disease and drug targets.

Venny 2.1 (https://bioinfogp.cnb.csic.es/tools/venny/), an online program, was utilized to visualize and isolate overlapping targets between the active ZSYTP compounds and those related to PCOS. The data were imported into Cytoscape 3.8.2 software to construct the active compound-potential target network (C-T network). The nodes represent the target genes and compounds, and the line edges represent the correlations between them. The size and transparency of the nodes were set to reflect the degree value. The active compounds and target with the highest degrees were selected for future molecular docking analysis.

PCOS disease targets and ZSYTP drug targets were imported into Cytoscape 3.8.2 software Biogenet add-in [20] to obtain Protein-Protein Interaction (PPI) networks, respectively. Both PPI networks were intersected for further topological screening.

#### 2.2.2. Gene Ontology and KEGG Enrichment Analysis

Cytoscape 3.8.2 software ClueGO 2.5.7 plug-in [21] was used to perform gene ontology (GO) enrichment analysis and Kyoto Encyclopedia of Genes and Genomes (KEGG) pathway enrichment analysis. Species was set as “*Homo sapiens,*” and a *p*-value <0.05 was set as statistically significant.

### 2.3. Confirmation of Molecular Docking

Molecular docking simulations were utilized to verify the binding of the target and the corresponding compound. Data about the construction of the macromolecular protein target receptors, including the first eight high degree values which come from the C-T network, were acquired via the RCSB PDB database [22] (PDB, http://www.rcsb.org/), and the small-molecule compounds which are considered as part of the first five high degree value coming from C-T network were retrieved via the PubChem database and TCMSP. Expulsion of water and ligand from macromolecular protein downloaded from PDB was performed using PyMOL 2.4 software [23], and format conversion was performed using Open Babel software [24]. Molecular docking simulations of the macromolecular protein target and the corresponding compounds were carried out by AutoDockTool 1.5.6 and AutoDock 4.2.6 software [25]. The genetic algorithm of the search parameters was used. Visualizing the results was utilized by PyMOL 2.4 software.

**Figure 1:**
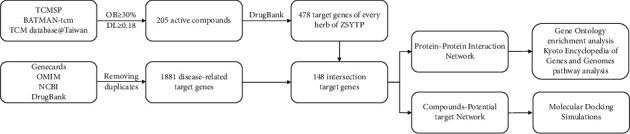
Workflow of the present study.

The workflow of our study is shown by flowchart (Figure 1).

## 3. Results

### 3.1. Screened Chemical Compounds of Each Herb in ZSYTP

1364 chemical compounds in ZSYTP were found in this process; there are 190 from *Panax ginseng* (RS), 188 from *Lycii Fructus* (GQZ), 174 from *Morindae officinalis Radix* (BJT), 165 from *Amomum aurantiacum* (SR), 135 from *Folium Artemisiae Argyi* (AY), 134 from *Codonopsis Radix* (DS), 119 from *Eucommiae Cortex* (DZ), 76 from *Rehmanniae Radix Praeparata* (SDH), 55 from *Atractylodes Macrocephala Koidz* (BZ), 46 from *Herba Taxilli* (SJS), 30 from *Dipsaci Radix* (XD), 29 from *Cuscutae Semen* (TSZ), 17 from *Fallopia multiflora* (HSW), 4 from *Colla Corii Asini* (EJ), and 2 from *Cornu Cervi Degelatinum* (LJS).

Upon application of ADME screening thresholds of OB ≥ 30% and DL ≥ 0.18%, 187 chemical compounds were obtained: 22 from RS, 45 from GQZ, 20 from BJT, 10 from SR, 9 from AY, 21 from DS, 28 from DZ, 2 from SDH, 9 from BZ, 2 from SJS, 6 from XD, 11 from TSZ, and 2 from HSW. LJS and EJ were not found in any online TCM databases; however, further 18 compounds with lower OB or DL values were consolidated as they hold extensive pharmacological activities, 10 from HSW, 4 from EJ, and 2 from LJS. These 205 compounds were considered as active compounds with good biological activity.

### 3.2. Active Target Genes Prediction of ZSYTP

205 active compounds were based on molecular similarity to predict the target genes, which was conducted by importing each unique molecular data into STITCH, Swiss Target Prediction, PubChem, and DrugBank. 478 target genes of every herb of ZSYTP were acquired after eliminating duplication.

### 3.3. Intersection Targets of ZSYTP and PCOS

**Figure 2:**
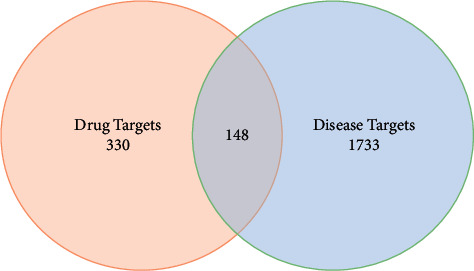
The Venn diagram of intersection potential target genes between drug and disease targets.

A total of 1881 PCOS disease-related targets were obtained upon removing duplicates. Further comparison was made between the 478 potential target genes of ZSYTP and 1881 PCOS disease targets; 148 intersection potential target genes that act on PCOS (Figure 2) were subsequently isolated. Of note, while there are multiple common targets between each herb to disease, target genes are known to have multipathway effects.

### 3.4. Constructed ZSYTP Compound-Potential Target Network (C-T Network)

C-T network illustrates the relationship among each herb, its corresponding active compounds, and targets. These were mapped out by the Cytoscape 3.8.2 software. The nodes describe herbs, active compounds, and target genes, and the edges indicate the relevance among them. A C-T network can provide further insights into the study of ZSYTP's therapeutic mechanism on PCOS.

**Figure 3:**
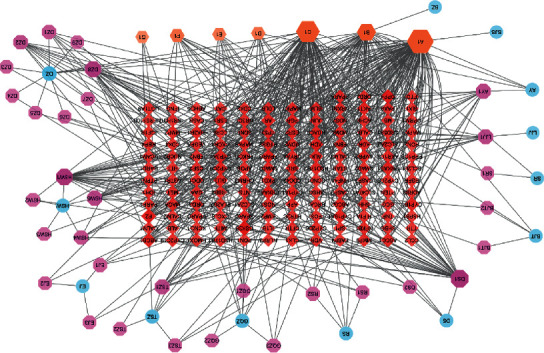
Compound-potential target network. Each blue ellipse represents a herb in ZSYT, and the pink octagons represent active compounds. The red diamonds represent the potential targets, and the orange hexagons at the bottom represent common active compounds. The size and transparency of each node are proportional to the target degree in the network.

The constructed C-T network of 39 active compounds includes 200 nodes and 540 edges depicted in Figure 3.

Compounds and targets with the highest degree values were utilized to do molecular docking verification. The degree values are listed in Table 1.

### 3.5. Core Network Analysis

PPI network of the intersectional potential target genes between disease and drug targets was constructed with Cytoscape version 3.8.2 BisoGenet plug-in.

**Figure 4:**
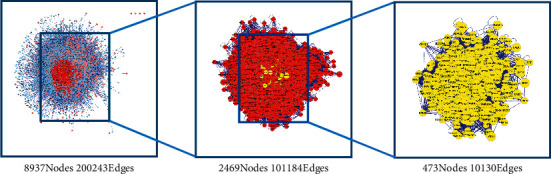
Topological screening process of PPI network.

8937 nodes and 200243 edges were established. Based on a previous study, topological feature analysis targets were selected with parameters above twice the median value. Upon the first screening process, a network of 2469 nodes and 101184 edges was derived. The second selection criteria were based on “Betweenness,” “Closeness,” “LAC,” and “Neighborhood Connectivity”; 473 nodes and 10130 edges were identified. The topological screening process is presented in Figure 4.

### 3.6. Gene Ontology and KEGG Analysis

**Figure 5:**
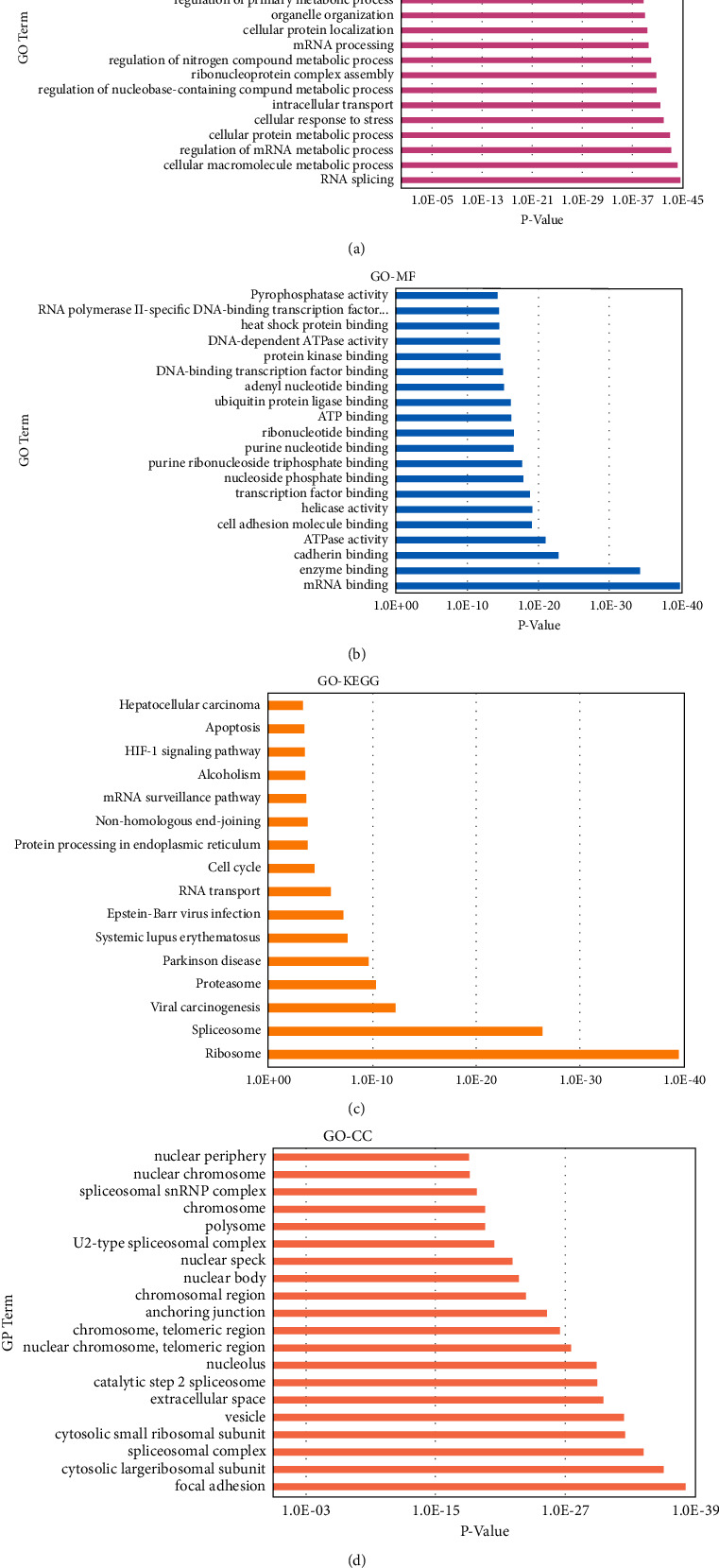
The GO function and KEGG pathway enrichments. (a) Enriched BP functions of active target genes. (b) Enriched MF functions of active target genes. (c) Enriched CC functions of active target genes. (d) KEGG pathway enrichment. BP: biological process; MF: molecular function; CC: cellular component; KEGG: Kyoto Encyclopedia of Genes and Genomes.

Cytoscape 3.8.2 software ClueGO plug-in was utilized to explore the active process of ZSYTP on PCOS by the 473 relationship genes from the PPI network, and *p* values less than 0.05 were defined as statistically significant. The visualization in this part is shown in Figure 5.

#### 3.6.1. Gene Ontology Biological Process (GO-BP)

The enrichment analysis of BP was completed with the Cytoscape ClueGO plug-in for visualization (Figure 5(a)), with *p*-values less than 0.05 as statistically significant. 540 processes were obtained. The top 20 biological processes with ascending *p*-values were selected for further analysis.

The biological processes selected were mainly involved in RNA splicing, cellular macromolecule metabolic process, regulation of mRNA metabolic process, cellular protein metabolic process, cellular response to stress, intracellular transport, regulation of the nucleobase-containing compound metabolic process, and regulation of nitrogen compound metabolic process.

#### 3.6.2. Gene Ontology Molecular Function (GO-MF)

107 terms were retrieved in gene ontology analysis of MF. Twenty molecular functions with the ascending *p* values were selected for further analysis and visualized in Figure 5(b). Gene ontology analysis of molecular function highlighted mRNA binding, enzyme binding, and cadherin binding as functions with the decreasing *p* value.

#### 3.6.3. Gene Ontology Cellular Component (GO-CC)

114 components were identified in gene ontology analysis of CC, including focal adhesion, cytosolic large ribosomal subunit, spliceosomal complex, cytosolic small ribosomal subunit, vesicle, extracellular space, catalytic Step 2 spliceosome, nucleolus, nuclear chromosome, chromosome, anchoring junction, chromosomal region, nuclear body, nuclear speck, polysome, and nuclear periphery. Twenty cellular components with ascending *p* values were selected for further analysis and visualized in Figure 5(c).

#### 3.6.4. KEGG Pathway Analysis

**Figure 6:**
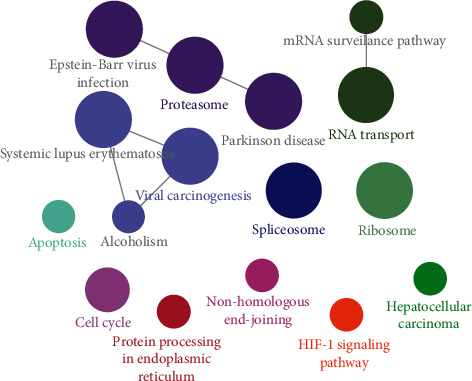
KEGG pathway enrichment analysis visualized by ClueGO.

Further KEGG analysis highlighted 16 pathways of ZSYTP potential pharmacological mechanisms on PCOS. All pathways are presented in Figure 5(d), and further visualization of the KEGG pathway is shown in Figure 6.

### 3.7. Molecular Docking Visualization

The lowest binding energy of molecular docking of potential targets and their appointed compounds is shown in Table 2 and the information of potential targets is shown in Table 3. In accordance with previous research, the binding activity of molecular docking simulations was considered that it is practicable when the binding energy was lower than −1.2 kcal/mol (−5 kJ/mol), and it is dynamite when the binding energy was lower than −5.0 kcal/mol. As presented in Table 2, all of the binding energy between the macromolecule and small-molecule is lower than −1.2 kcal/mol. 23.3% of the binding energy is lower than −5.0 kcal/mol. Furthermore, after molecular docking, the potential targets with their original ligands and the comparison of the lowest binding energy between potential targets-drug compounds and the targets-original ligands showed that the drug compounds were showing potential to be the active compounds of the potential targets in vivo.

**Figure 7:**
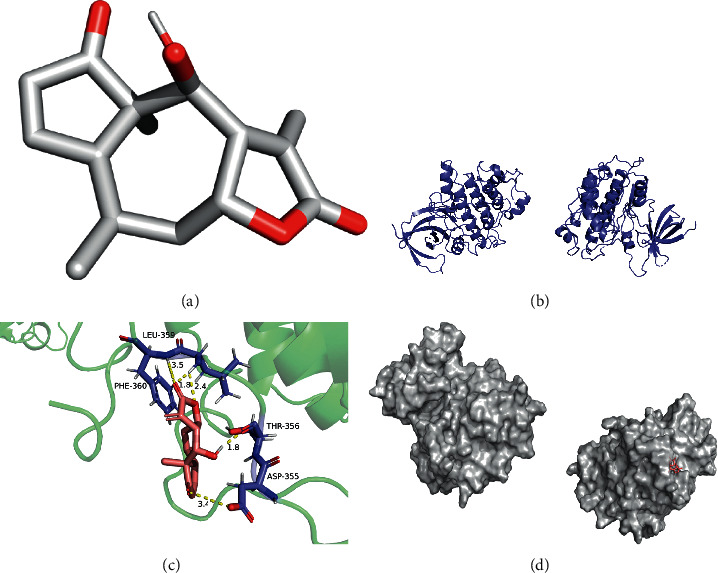
GSK3B helenalin molecular docking. 3D structures of (a) helenalin and (b) GSK3B. (c) Molecular docking simulation and (d) display protein surface of molecular docking simulation.

**Figure 8:**
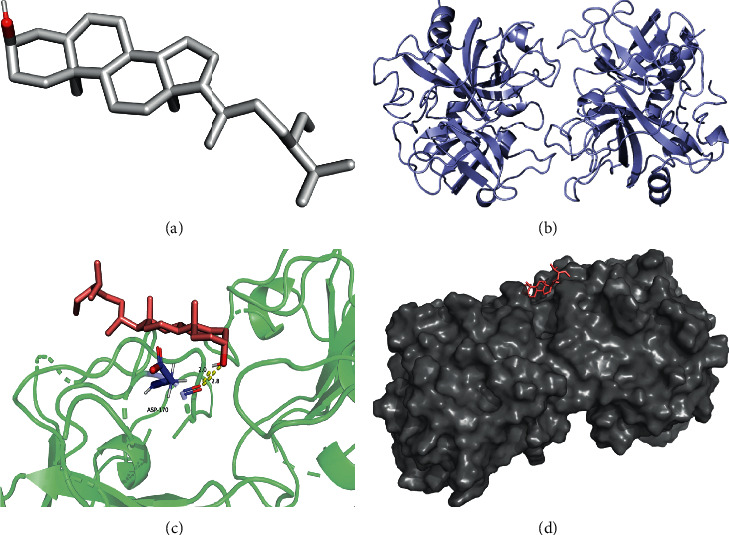
F2 beta-sitosterol molecular docking. 3D structures of (a) beta-sitosterol and (b) F2. (c) Molecular docking simulation and (d) display protein surface of molecular docking simulation.

**Figure 9:**
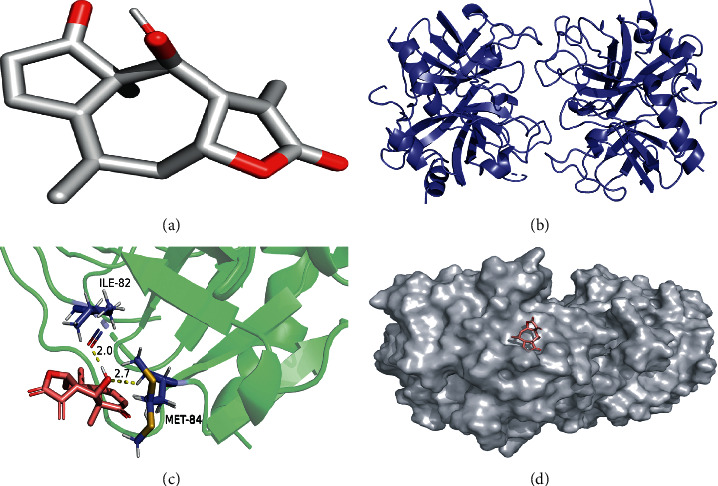
F2 helenalin molecular docking. 3D structures of (a) helenalin and (b) F2. (c) Molecular docking simulation and (d) display protein surface of molecular docking simulation.

**Figure 10:**
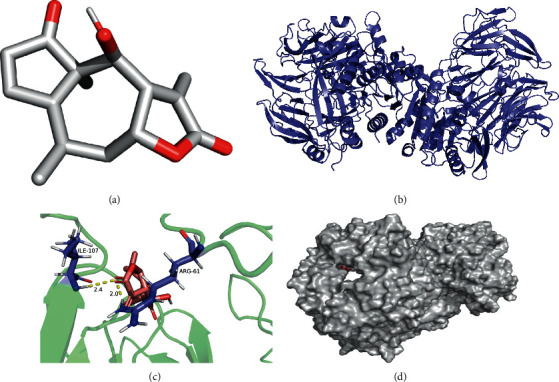
DPP4 helenalin molecular docking. 3D structures of (a) helenalin and (b) DPP4. (c) Molecular docking simulation and (d) display protein surface of molecular docking simulation.

**Figure 11:**
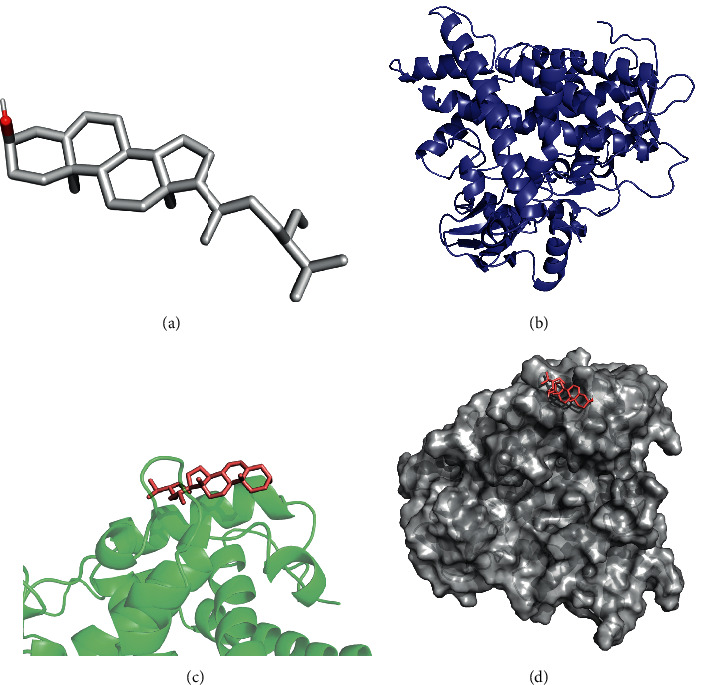
CYP1B1 beta-sitosterol molecular docking. 3D structures of (a) beta-sitosterol and (b) CYP1B1. (c) Molecular docking simulation and (d) display protein surface of molecular docking simulation.

**Figure 12:**
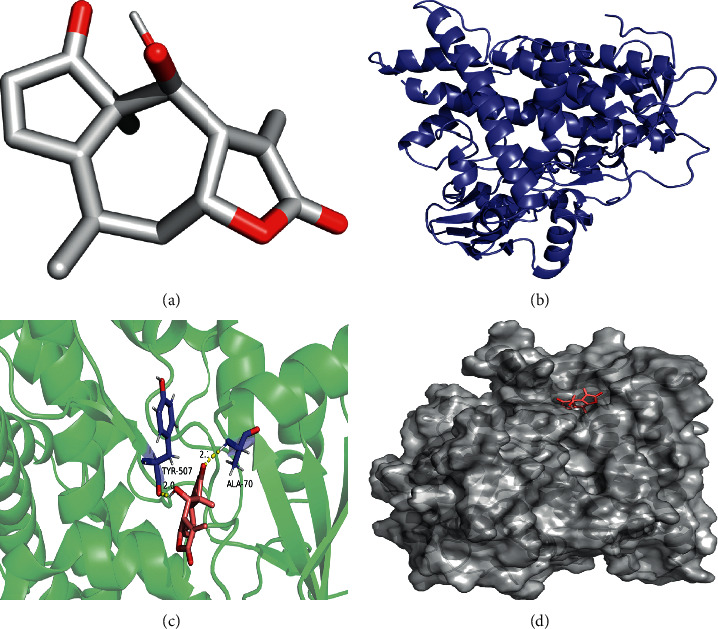
CYP1B1 helenalin molecular docking. 3D structures of (a) helenalin and (b) 3D structures of CYP1B1. (c) Molecular docking simulation and (d) display protein surface of molecular docking simulation.

The molecular docking simulations of GSK3B helenalin, F2 beta-sitosterol, F2 helenalin, DPP4 helenalin, CYP1B1 beta-sitosterol, and CYP1B1-helenalin are shown in Figures 7–12, respectively.

These figures show the residues and hydrogen bonds that the ligand binds to the protein. The residues are indicated in bluish violet, while the hydrogen bonds are dotted in yellow.

Visualization of beta-sitosterol in *Lycii Fructus*, *Cuscutae Semen*, *Eucommiae Cortex*, *Artemisiae argyi*, *Panax ginseng*, *Morindae officinalis Radix*, and *Amomum aurantiacum* and helenalin in *Eucommiae Cortex* among the disease targets is shown to be stable and feasible. Therefore, these may warrant more research for further validation of the therapeutic effects of ZSYTP.

## 4. Discussion

ZSYTP was first used to prevent recurrent spontaneous and threatened abortion. In line with TCM's theory of treating different diseases with the same therapeutic principle and on the basis that the kidney in TCM's theory is closely related to reproduction, ZSYTP is also used to treat irregular menstruation and infertility in clinical practice, such as PCOS [26–30].

It has been found that ZSYTP can improve the clinical symptoms of PCOS patients [5]. Therefore, our team sought to elucidate ZSYTP's pharmacological mechanism on PCOS.

In order to further support the existing literature and provide insights and possible hypotheses for the pharmacological mechanism of ZSYTP in the treatment of PCOS, we conducted extensive literature research on the active compounds as highlighted by the PPI networks and 16 pathways as indicated by KEGG pathway enrichment analysis.

### 4.1. Regulation of HIF-1 Expression Levels and Ovulation

As a tonifying kidney TCM formula, ZSYTP is found to have therapeutic effects in the treatment of POI and PCOS [31]. However, the pharmacological mechanisms of ZSYTP remain unclear.

Our team conducted a network pharmacology study on the effect of ZSYTP on POI. One hypothesis put forward by our previous study was that ZSYTP upregulates the hypoxia-inducible factor-1 (HIF1) signaling pathway [32], thereby encouraging oocyte competence through a multitude of pathways. Similarly, the KEGG analysis of ZSYTP on PCOS highlighted the HIF-1 pathway, indicating a possible relationship between the treatment efficacy of ZSYTP and this pathway.

HIF-1 is a transcription factor, and its activity is mainly determined by HIF-1*α*, which is regulated by the low oxygen concentration of the environment [33]. Human oocytes grow in follicular fluid surrounded by a basement membrane, where there is a lack of blood supply [34] and a low oxygen concentration of about 1.3%–5.5% in the human follicular fluid [35]. Development of the follicle, subsequent maturation, and ovulation all through to the early luteal phase takes place in a hypoxic environment [36].

One study showed that polycystic ovaries were found in rats when submitted into hypobaric hypoxia. Weight of the ovaries increased under a hypoxic environment, while synthesis and secretion of E2 were negligible [37].

Multiple animal experimental studies suggest that HIF-1 promotes follicular development and interacts with FSH receptors on granulosa cells to regulate E2 secretion [38]. One other study found high expressions of HIF-1 mRNA in dominant follicles and negligible amounts of HIF-1 mRNA expression in preatretic follicles, suggesting that HIF-1 expression might be crucial in follicular development [39]. This is further substantiated by another study investigating the expression patterns of HIF-1a in endometrial tissues of PCOS and healthy women. The study found that mRNA and HIF-1*α* protein expression in PCOS women were significantly lower than those of the control group, indicating HIF-1*α*′s possible involvement in the molecular mechanisms of endometrial dysfunction in PCOS women, affecting fertility [40].

The balance between HIF-1 expression and oxygen concentration of the environment is therefore crucial for normal follicle development and subsequent ovulation to take place.

Quercetin is an active compound in ZSYTP with the highest degree, as indicated in the Compound-Potential Target Network (Figure 3). It has been widely reported that hypoxia is the only known naturally occurring signal to activate the HIF-1 transcription factor. A recent study concluded that quercetin activates HIF-1*α* in a manner similar to hypoxia and stabilizes HIF-1*α* [41]. Quercetin is also found to reduce high testosterone levels, luteinizing hormone (LH), and insulin resistance in PCOS. Multiple studies have postulated that the benefits of quercetin could be due to its antioxidant and anti-inflammatory properties and concluded that quercetin could be considered as a possible treatment for PCOS [42].

It is possible that the active compounds in ZSYTP like quercetin activate the HIF-1 transcription factor, promoting follicular development in PCOS patients and thereby reducing follicular atresia and regulating ovulation.

### 4.2. Insulin Resistance and Ovarian Function

PCOS women have an increased risk of insulin resistance (IR), while multiple studies have shown that IR plays an important role in the occurrence and development of PCOS as well [43]. Due to impaired use of glucose by peripheral tissues, the pancreas secretes more insulin in response to the increased glucose levels in the blood. This process involves the activation of the phosphatidylinositol-3-phosphate kinase (PI3K) and protein kinase B (AKT) pathway [44].

When PI3K is activated, it affects the intracellular glucose transporter (GLUT) to transport glucose across the membrane. Studies have shown that GLUT4 expression in the PCOS uterus is significantly reduced, thus affecting glucose metabolism in the body. Quercetin, the active ingredient in ZSYTP, can increase the expression of this gene [45]. In addition to improving ovarian function, it can also improve endometrial function and inflammatory microenvironment by regulating IR state [46]. At the same time, androgen secretion can be decreased by downregulating the expression of the CYP17A1 gene [47].

Normally, all female follicles develop through a series of processes such as recruitment, selection, dominance, and ovulation, in which physiological concentrations of insulin levels promote granulosa cell proliferation, and glucose metabolism in granulosa cells provides energy for oocyte development, thereby regulating follicular development. In addition, the insulin signaling pathway affects ovarian physiological function, oocyte developmental quality, and the ovulation process [48].

In the PPI network, through the analysis of the active components and targets of ZSYTP, it is predicted that the main active components and targets of ZSYTP have a good potential to improve IR in PCOS. Not only quercetin but also luteolin and emodin have a multidirectional regulation effect on the etiology of PCOS that has been explored [49].

Luteolin and emodin can improve the environment of high insulin levels by promoting PI3K/AKT pathway [50, 51] and thus upregulating CYP1B1 gene expression, which is a potential target gene of ZSYTP and PCOS, and CYP1B1 is involved in steroid and lipogenesis and metabolism [52] and also relates to improving the aromatase activity of ovarian granulosa cells in an environment of high insulin levels, which enhanced expression of FSH receptors and increased estrogen produciton. The sensitivity of granulosa cells to FSH was increased, thereby improving follicular dysgenesis [53], which in turn reduces the number of atretic follicles and restores the motility cycle.

Dipeptidyl peptidase-4 (DPP4) is one of the main potential target genes of ZSYTP and PCOS, and studies have shown that it plays a key role in the regulation of insulin secretion. DPP4 serum activity is elevated in patients with polycystic ovary syndrome, DPP4 serum levels are closely related to anti-Müllerian hormone (AMH) serum levels [54], and the use of DPP4 inhibitors may improve IR [55].

Patients with PCOS often have symptoms of ovulatory disturbance or anovulation, which may be related to their IR, and our results from molecular docking suggest that ZSYTP may improve IR associated with PCOS, thus directly or indirectly improving ovarian function.

### 4.3. Relation between Epigenetics and PCOS

Gene expression in eukaryotes is performed by RNA polymerase, spliceosome, and ribosome, respectively [56], and KEGG pathway enrichment analysis in the study shows that the mechanism of ZSYTP in treating PCOS was closely related to these dynamic molecular machines. PCOS patients have the characteristics of family aggregation and heterogeneity [57], which also suggests that PCOS is closely related to the combined effects of genetics and environment. An abnormal epigenetic modification is closely related to the occurrence and inheritance of multiple diseases, which may be the medium between environmental factors and human diseases [58]. Moreover, with different changes in the gene nucleotide sequence, epigenetic changes are often reversible [59], which provides a new thought for the mechanism study of ZSYTP in the treatment of PCOS.

## 5. Conclusion

The pathogenesis of PCOS still remains undefined; therefore, there is no effective cure for this disease. Although most fertility problems in PCOS patients can be resolved through symptomatic treatment, PCOS still severely affects women's long-term health.

Hence, this paper aims to investigate the possible etiology of PCOS and explore the pharmacological mechanism between ZSYTP and PCOS. We extracted 205 active compounds from ZSYTP and simultaneously retrieved 1881 disease target genes on multiple databases. 148 intersection target genes were derived by intersecting the databases of disease and drug-target genes.

Subsequently, our team constructed C-T network and multiple PPI networks and performed the GO and KEGG analysis. This study hypothesizes that ZSYTP may alleviate IR to treat PCOS. GO and KEGG analysis also highlighted that the HIF-1 signaling pathway and gene expression could be the pathological mechanisms of PCOS.

Additionally, the result of this research shows that the same compounds in ZSYTP could regulate multiple targets; meanwhile, the same targets could intervene in multiple biological processes and pathways.

It reflects the characteristics of the combined action of multipathway and multitarget of ZSYTP. The results suggested that the whole network can be regulated by regulating single or multiple targets in the network, which provides a scientific basis and new insights for the clinical application of ZSYTP in the treatment of PCOS and simultaneously provides a new direction for further exploring the potential mechanism of ZSYTP on PCOS.

This study is mainly based on bioinformatics and massive data calculation results, which will be further screened and verified by animal or cell experiments to clarify the main regulatory targets of ZSYTP, thus promoting the development of modern pharmacology of TCM.

## Figures and Tables

**Table 1:** List of top six active compounds of ZSYTP based on degree.

Active compound	Degree	Herbs involved
Quercetin	125	GQZ, TSZ, DZ, AY, and SJS
Kaempferol	96	TSZ, DZ, and RS
Beta-Sitosterol	54	GQZ, TSZ, DZ, AY, RS, BJT, SR, HSW, and BZ
Luteolin	35	DS
Emodin	26	HSW
Helenalin	23	DZ

**Table 2:** Lowest binding energy of compounds-target molecular docking.

Target	Degree value	Original ligands	Quercetin	Kaempferol	Beta-Sitosterol	Luteolin	Emodin	Helenalin
GSK3B	19	−3.61	−1.86	−2.5	−4.31	−2.37	−1.53	−5.02
F2	15	−2.06	−2.97	−4.82	−6.67	−4.48	−4.62	−6.95
ABCB1	13	−2.33	−1.76	−2.28	−4.14	−2.04	−3.57	−4.57
DPP4	13	−2.19	−2.51	−2.64	−4.36	−2.39	−3.97	−5.72
CYP1B1	12	−6.43	−2.99	−3.76	−5.72	−4.96	−4.6	−6.33

GSK3B: glycogen synthase kinase 3 beta; F2: coagulation factor II; ABCB1: ATP binding cassette subfamily B member 1; DPP4: dipeptidyl peptidase 4; CYP1B1: cytochrome P450 1B1.

**Table 3:** The information of potential targets.

Protein name	Gene name	PDB ID
Glycogen synthase kinase 3 beta	GSK3B	3F88
Coagulation factor II, thrombin	F2	2AFQ
ATP binding cassette subfamily B member 1	ABCB1	7A69
Dipeptidyl peptidase 4	DPP4	6B1E
Cytochrome P450 1B1	CYP1B1	3PM0

## Data Availability

The datasets supporting the conclusions of this article are available in a public database from TCMSP, TCM Database@ Taiwan, BATMAN-TCM, Swiss Target Prediction, STITCH, PubChem, DrugBank, UniProt, GeneCards, OMIM, NCBI, and RCSB PDB.

## Abbreviations

RS: *Panax ginseng*
GQZ: *Lycii Fructus*
BJT: *Morindae officinalis Radix*
SR: *Amomum aurantiacum*
AY: *Folium artemisiae Argyi*
DS: *Codonopsis Radix*
DZ: *Eucommiae Cortex*
SDH: *Rehmanniae Radix Praeparata*
BZ: *Atractylodes macrocephala Koidz*
SJS: *Herba Taxilli*
XD: *Dipsaci Radi*x
TSZ: *Cuscutae Semen*
HSW: *Fallopia multiflora*
EJ: *Colla Corii Asini*
LJS: *Cornu Cervi Degelatinum*
OB: Oral bioavailability
DL: Drug-likeness
TCM: Traditional Chinese medicine
ZSYTP: Zishen Yutai Pills
PCOS: Polycystic ovary syndrome
ADME: Adsorption, Distribution, Metabolism, Excretion
HIF-1: Hypoxia-inducible factor 1
IR: Insulin resistance.
